# Putative mutations associated with tetracycline resistance detected in Treponema spp.: an analysis of 4,355 Spirochaetales genomes

**DOI:** 10.1099/acmi.0.000963.v4

**Published:** 2025-10-13

**Authors:** Sheeba Santhini Manoharan-Basil, Zina Gestels, Saïd Abdellati, Tessa de Block, Thibaut Vanbaelen, Irith De Baetselier, Chris Kenyon

**Affiliations:** 1 STI Unit, Department of Clinical Sciences, Institute of Tropical Medicine Antwerp, 2000 Antwerp, Belgium; 2 Clinical and Reference Laboratory, Department of Clinical Sciences, Institute of Tropical Medicine, 2000 Antwerp, Belgium; 3 Department of Medicine, University of Cape Town, Cape Town 7700, South Africa

**Keywords:** doxycycline post-exposure prophylaxis (PEP), tetracycline resistance, *Treponema pallidum*

## Abstract

The resurgence of syphilis has necessitated novel prophylactic strategies, such as the use of doxycycline post-exposure prophylaxis. However, the potential for increased doxycycline use to select for tetracycline resistance represents significant challenges in managing this sexually transmitted infection. This study aims to identify chromosomal mutations associated with tetracycline resistance in *Spirochaetales* to inform molecular surveillance tools. Whole-genome sequences (WGSs) from the *Spirochaetales* order, including 4,355 genomes, were analysed for the presence of mutations in 16S rRNA and non-synonymous mutations in the *rpsC* and *rpsJ* genes. The study utilized WGS from GenBank® and sequence data from the PubMLST *Treponema pallidum* isolate collection. Genetic resistance to tetracycline was detected using a combination of blastn searches and gene–gene analysis. A transition mutation TGA to TGG at positions 965–967 in the 16S rRNA gene was detected in 5.6% of *Treponema* spp. and 4.0% of *Spirochaeta* spp. genomes. The *rpsJ* gene exhibited a V57G aa substitution across a significant subset of *Treponema* spp. (*n*=14) and *Spirochaeta* spp. (*n*=1). Notably, the V57K substitution was present in *Spirochaeta* spp. (*n*=17) and *Treponema* spp. (*n*=15). The *rpsC* gene had the H178Q mutation and was found to be present in the *Spirochaetales* bacterium (*n*=4). The identification of putative mutations associated with tetracycline resistance in *Spirochaetales* provides a foundation for the development of rapid molecular tests. This study underscores the complexity of antibiotic resistance mechanisms and the critical importance of surveillance of genetic resistance determinants in the era of antibiotic prophylaxis for sexually transmitted infection management.

## Data Summary

Tables S1 to S4 (available in the online Supplementary Material) and the 16SrRNA, *rpsC* and *rpsJ* multiple alignment files along with custom bash scripts for (1) rRNA gene detection and quantification using Barrnap; (2) genome quality assessment using CheckM; (3) gene-by-gene schema creation, allele calling and cgMLST extraction using chewBBACA; and (4) extraction of rRNA copy number per genome have been deposited and are accessible at the following link: https://zenodo.org/records/15753527.

## Introduction

The incidence of bacterial sexually transmitted infections in general, but syphilis in particular, has recently increased dramatically in the world, including Europe [1]. These increases have led to calls for novel measures to reverse this trend. One of the most promising interventions is the ingestion of doxycycline after condomless sex [doxycycline post-exposure prophylaxis (doxy-PEP)]. Doxy-PEP has been shown to reduce the incidence of syphilis, chlamydia and possibly gonorrhoea in men who have sex with men (MSM) and transgender women [2,4]. These findings have led a number of organizations, such as the U.S. Centers for Disease Control and Prevention (CDC) and the European AIDS Clinicians Society (EACS), to promote the use of doxy-PEP in sub-populations of MSM [5]. We have calculated that the introduction of doxy-PEP in Belgian HIV pre-exposure prophylaxis cohorts would increase the consumption of doxycycline by up to 88-fold [6]. This intense consumption of doxycycline could induce and select for antimicrobial resistance (AMR) to tetracyclines and other antimicrobials in a range of bacterial species [7,9]. Doxycycline is a vital backup medication for people with syphilis who cannot receive intramuscular penicillin due to frequent stock-outs, silicone implants or penicillin allergy [10]. If doxy-PEP were to induce tetracycline resistance in *Treponema pallidum* subsp. *pallidum*, the causative agent of syphilis, this would have far-reaching consequences.

Previous genotypic assessments of *T. pallidum* have not found mutations that are predicted to cause tetracycline resistance [11,12]. One exception is a study from China that detected the *tetB* gene directly from 15/171 syphilitic lesions via PCR amplification [13]. Concerns have, however, been raised that the *tetB* genes detected represented contamination from other bacteria present in these clinical samples [14]. Because *T. pallidum* is not known to contain any mobile elements, point mutations in specific regions of the gene encoding the 16S rRNA or specific ribosomal proteins would be the most plausible pathway to tetracycline resistance [12,15]. Furthermore, A2058G and A2059G mutations in *T. pallidum*’s 23S rRNA that confer resistance to macrolides have arisen independently in multiple lineages of *T. pallidum* and spread to attain prevalences close to 100% [16,17]. *T. pallidum* genome contains two copies of the rRNA (16S-23S rRNA-5S) that are part of the single operon*,* and thus, point mutations are possible in the 16S rRNA gene [18].

Mutations in the 16S rRNA gene have been implicated in tetracycline resistance across a range of bacterial species, including *Propionibacterium acnes*, *Helicobacter pylori*, *Mycoplasma bovis* and *Streptococcus pneumoniae* [19]. In *H. pylori*, a triple mutation at positions 965–967 (AGA→TTC) within the h31 loop and a deletion at position G942 (*Escherichia coli* numbering) were each shown to confer tetracycline resistance [20]. These sites correspond to the Tet-1 primary binding site and the Tet-4 secondary binding site, respectively, on the 16S rRNA [21,22]. Likewise, mutations in chromosomal genes encoding ribosomal proteins, such as *rpsC* (ribosomal protein S3) and *rpsJ* (ribosomal protein S10), have been implicated in reduced susceptibility to tetracyclines and related antibiotics [19]. In *S. pneumoniae*, aa substitutions Lys4Arg and His175Asp in RpsC have been associated with reduced tigecycline susceptibility, and functional studies have shown that ribosomal protein S3 plays a critical role in tetracycline binding [23,24]. Similarly, substitutions at Val57 (Lav57Leu) in RpsJ have shown decreased susceptibility to tetracyclines in multiple species, including *Klebsiella pneumoniae* [25]. Thus, substitutions in RpsC and RpsJ in the context of tetracycline resistance were explored across the *Spirochaetales*.

*T. pallidum* is challenging to culture *in vitro*, and, thus far, studies that have established the doxycycline MICs of the strains assessed were between 0.06 and 0.1 mg l^−1^ [26,28]. A recent study attempted to induce tetracycline resistance *in vitro* by exposing the SS14 strain both intermittently and continuously to sub-bactericidal doxycycline concentration and found that no resistance developed [29]. No study has, however, attempted to induce tetracycline resistance *in vivo*. For such studies and for the evaluation of suspected tetracycline resistance in human infections, it would be useful to know which resistance mutations are possible based on what resistance-associated mutations occur in related bacterial species. Thus, in this study, we used the genomes from the *Spirochaetales* order as this broader scope allows for a more comprehensive understanding of resistance mechanisms across related species, offering insights into horizontal gene transfer, variations in genetic pathways and the evolution of tetracycline resistance beyond a single species. This *in silico* study assessed the occurrence and prevalence of tetracycline resistance associated with chromosomal mutations in the order *Spirochaetales*. The *Spirochaetales* order contains three families: *Spirochaetaceae*, *Brachyspiraceae* and *Leptospiraceae*. Previous studies have found that mutations in 16S rRNA, *rpsC* and *rpsJ* have been shown to be associated with decreased susceptibility to tetracycline in several other spirochaetes, such as *Borrelia* spp. and *Leptospira* spp. [30,32].

## Methods

### Dataset for analysis

**Fig. 1.**
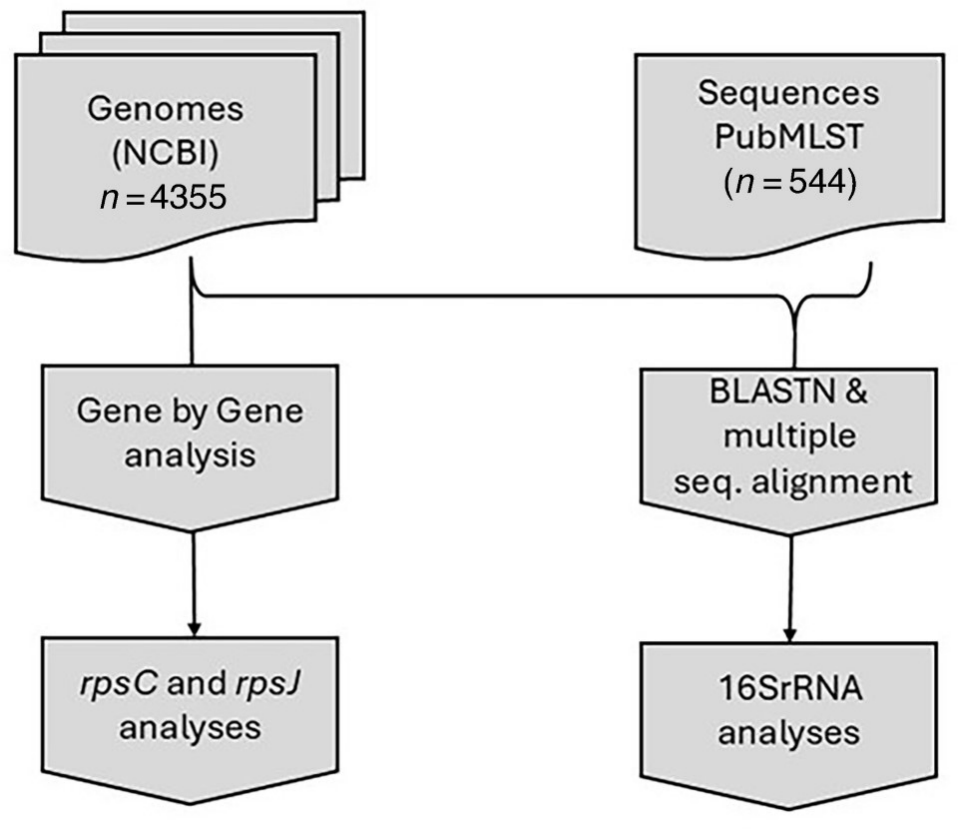
Flowchart describing genomes and sequences used in the analyses.

The whole-genome sequences (WGSs) used in this study were acquired from GenBank® (assessed on 15 February 2024). This collection included 4,355 genomes from the order *Spirochaetales* (NCBI Taxonomy ID: 136). Genome completeness and contamination were evaluated using CheckM v1.2.2. Notable species include *Treponema* sp., with 614 genomes, and *T. pallidum*, accounting for 417 genomes. Additionally, *Borreliella burgdorferi* (Lyme disease spirochete) and uncultured *Treponema* species were well represented, with 445 and 559 genomes, respectively. For analytical clarity, the genomes were grouped by species and subspecies-level classifications (Tables S1 and S2). These included 445 genomes classified as Lyme disease spirochetes, 958 as *Spirochaeta* spp., 881 as other *Treponema* spp. (including *Treponema socranskii*, *Treponema phagedenis* and *Treponema brennaborense*, among others), 96 as *T. pallidum* subsp. *pallidum* (syphilis treponeme) and 25 as *T. pallidum* subsp. *pertenue* (yaws treponeme). Furthermore, to assess 16S rRNA gene copy number, we used the BAsic Rapid Ribosomal RNA Predictor (Barrnap v0.9) (https://github.com/tseemann/barrnap). Copy number information is provided in Table S2. Additionally, the study incorporated full-length 16S sequence data from the PubMLST *T. pallidum* isolate collection, where a single 16S rRNA copy per isolate was available (Table S3). This collection included 544 isolate sequences. For 16S, analysis of mutations at positions 965–967 (*E. coli* numbering) was prioritized for reporting based on experimental evidence linking these sites to tetracycline resistance in *H. pylori* [20]. Similarly, substitutions such as Lys4Arg and His175Asp in RpsC and Val57Leu in RpsJ were evaluated due to their known association with tetracycline and tigecycline resistance [24,25]. A flowchart describing the genomes and sequences used in the analyses is provided in Fig. 1. The list of genomes used in this analysis is provided in Table S3.

### Detection of genetic resistance to tetracycline

WGSs were screened for the presence of mutations associated with tetracycline resistance, and the analysis was carried out as described by Manoharan-Basil *et al*. [33]. In brief, the WGSs (*n*= 4355) were analysed using chewBBACA v2.8.5 [34], which generates an output file that classifies the loci as follows: EXC (exact match), INF (inferred novel alleles), LNF (locus not found), PLNF (probable locus not found), PLOT3/PLOT5 (locus located at the contig tip), LOTSC (locus larger than contig), ASM/ALM (alleles smaller or larger than expected) and NIPH/NIPHEM (multiple hits or paralogous alleles). First, a training file was created from the complete genome of *T. pallidum* (AE000520.1) using Prodigal and used in subsequent steps [35]. Second, a study-specific *T. pallidum* schema was created, and a FASTA file for each CDS was generated. Third, a blast database was created using the study-specific schema, herein referred to as the schema database. This was followed by querying the target genes, *rpsC* and *rpsJ* genes from *T. pallidum* (AE000520.1), against the schema database using a blastn search. The multiple sequence alignment files were imported into CLC Genomics Workbench (v20), and the CDSs were translated. The presence of non-synonymous substitutions in the following proteins was further evaluated: RpsC (Lys4; His175) and RpsJ (Val57).

For 16S analysis, a blastn search using the 16S rRNA *T. pallidum* (AE000520.1) sequence was queried against the blast database that was created using all 4,355 genomes. A blastn search was also performed against *T. pallidum* isolate collection in PubMLST. Thus, for the 16S analysis, the dataset included the genomes and sequences from GenBank and PubMLST, respectively.

All scripts used in this study – including those for rRNA gene detection and copy number analysis (Barrnap), genome quality assessment (CheckM), and gene-by-gene analysis (chewBBACA) – are available in the Zenodo repository. These include .sh scripts for batch execution of Barrnap and CheckM, as well as schema creation, allele calling and cgMLST extraction using chewBBACA.

### Molecular typing using MLST scheme

MLST of the isolates was carried out using the TP0136 (putative outer membrane protein gene), TP0548 (putative membrane protein gene) and TP0705 (penicillin-binding protein gene) loci. The sequences were retrieved from the NCBI using the accession IDs provided in Grillová *et al*. [36]. A local database was created using the ‘schema seed’ output from chewBBACA, and the three genes were queried against this database using local blastn [37]. These loci were used as input for typing using the MLST (grillová) scheme as implemented in PubMLST within the *T. pallidum* typing database [38].

## Results

Gene availability of the 16S rRNA and *rpsC* and *rpsJ* loci varied across 4,355 genomes (Table 1). For the 16S rRNA gene, 1,925 loci were found (LF), and for 2,430 genomes, the locus was not detected. For the *rpsJ* gene, 3,326 detections (LF) were classified as exact or inferred matches from the genomes, followed by 995 genomes that did not have the locus. Similarly, for the *rpsC*, 2,386 loci were identified and 1,924 genomes did not have the *rpsC* gene. Further details of the locus assignments are provided in Table 1. Genome completeness and contamination were evaluated using CheckM v1.2.2 and are provided in Table S1.

**Table 1.** Locus classification summary for 16S rRNA and *rpsC* and *rpsJ* genes across 4,355 *Spirochaetales* genomes based on chewBBACA output

Locus summary	16S rRNA	*rpsC*	*rpsJ*
LF	1,925	2,386	3,326
ALM		14	3
ASM		1	1
LNF	2,430	1,924	995
NIPH		18	20
NIPHEM		1	
PAMA			8
PLOT3		8	
PLOT5		3	2

LF, locus found with either an exact match (EXC) or inferred allele (INF); LNF, locus not found – no sufficiently similar allele detected; ALM/ASM, allele larger (ALM) or smaller (ASM) than the expected size range for that locus; NIPH/NIPHEM, multiple hits within the same genome suggest possible paralogous loci (NIPH) or exact match paralogues (NIPHEM); PLOT3/PLOT5, locus located at the 3′ or 5′ end of a contig, potentially truncated due to genome fragmentation; PAMA, partial or ambiguous match.

### Mutations in the 16S rRNA gene

**Fig. 2.**
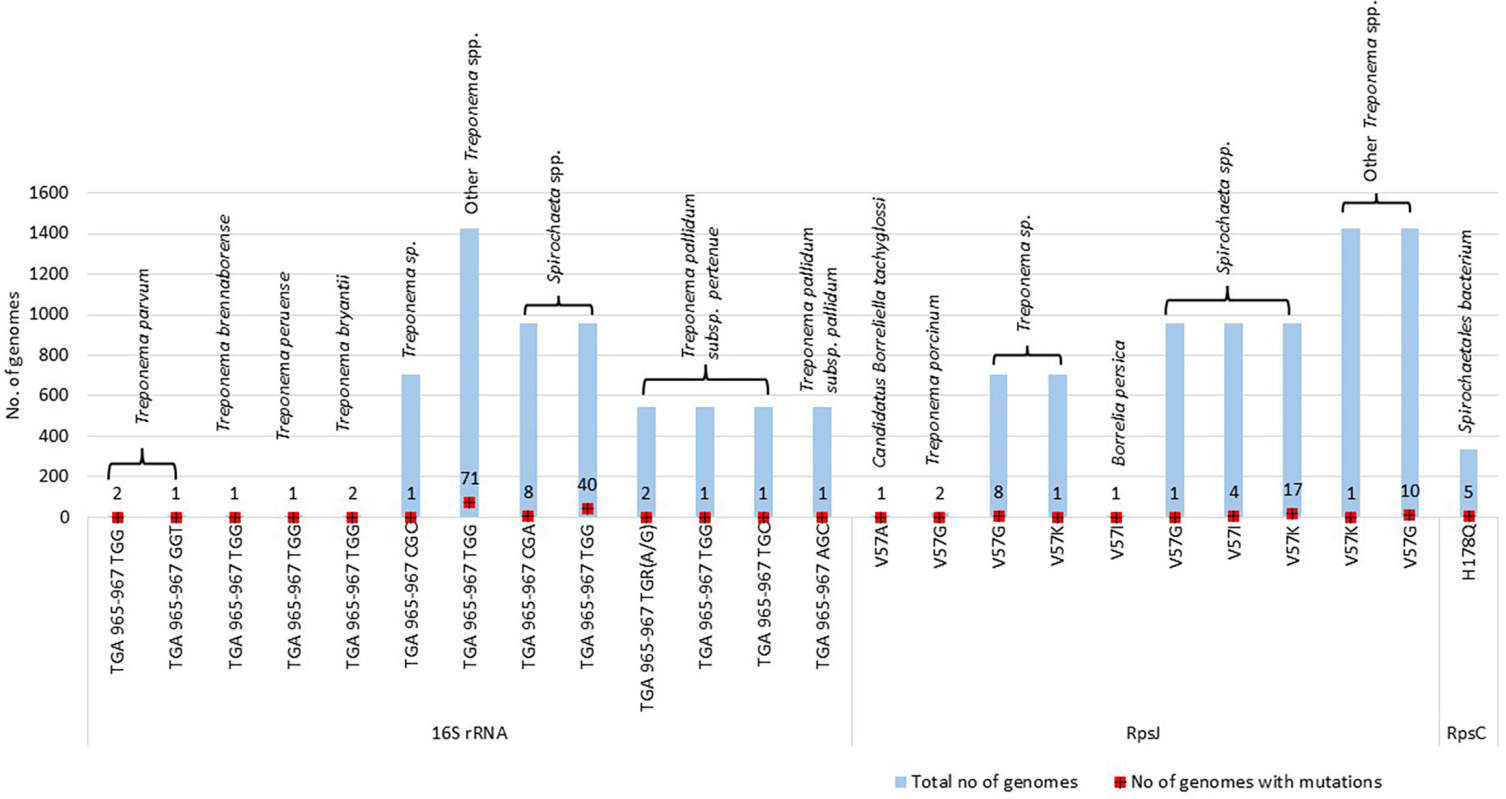
Bar graph showing the mutations detected in the 16S rRNA gene and substitutions in ribosomal proteins RpsJ (30S subunit protein S10) and RpsC (30S subunit protein S3). Light blue bars represent the total number of genomes analysed for each organism or group, while red squares indicate the number of genomes in which the corresponding mutation or substitution was detected. Mutation counts are annotated above each red square.

Our analyses revealed a transition mutation from TGA to TGG at positions 965 to 967 (*E. coli* 16S rRNA numbering) among *Treponema* (5.6% occurrence) and *Spirochaeta* spp. (4% occurrence) genomes (Fig. 2). Other mutations at the same positions, including CGC, GGT, CGA, TGR(A/G), TGC and AGC, were detected less frequently (Table 2 and Fig. 2). Among the five *T. pallidum* sequences from the PubMLST collection, mutations at positions 965–967 of the 16S rRNA gene included TGA to TGR (A/G), TGG, TGC and AGC, observed across isolates of *T. pallidum* subsp. *pertenue* and *pallidum* (Table 2 and Fig. 2). The multiple sequence alignment of the NCBI and PubMLST 16S rRNA sequences is provided as File S1a, b, respectively.

**Table 2.** List of the tetracycline resistance-associated mutations

Ribosomal gene/protein	Source of genomes	ID	Organism	Isolate	Country	Host species	Source	Clinical source	Total no. of genomes	Mutation	No. of genomes with mutations	No. of genomes with 2 copies	No. of genomes with >2 copies	Copy no.
16S rRNA	GenBank	–	*Treponema parvum*	–	–	–	–	–	5	TGA 965-967 TGG	2	2		1–2 copies
										TGA 965-967 GGT	1	0		1 copy
		–	*T. brennaborense*	–	–	–	–	–	4	TGA 965-967 TGG	1	1		4 copies
		–	*Treponema peruense*	–	–	–	–	–	4	TGA 965-967 TGG	1	1		4 copies
		–	*Treponema bryantii*	–	–	–	–	–	5	TGA 965-967 TGG	2	0	3	1–4 copies
		–	*Treponema* sp.	–	–	–	–	–	60	TGA 965-967 CGC	1	0		1 copy
		–	Other *Treponema* spp.	–	–	–	–	–	1341	TGA 965-967 TGG	71	24	9	1–3 copies
		–	*Spirochaeta* spp.	–	–	–	–	–	1,006	TGA 965-967 CGA	8	0		1 copy
		–								TGA 965-967 TGG	40	18		1 to 5 copies
	PubMLST	1584	*T. pallidum* subsp. *pertenue*	Oka_2116	Ivory Coast	*Cercocebus atys*	Clinically acquired	Genital swab	544	TGA 965-967 TGR(A/G)	2	–	–	–
		1585	*T. pallidum* subsp. *pertenue*	LMNP-2	Tanzania	*Papio anubis*	Clinically acquired	Genital swab				–	–	–
		1582	*T. pallidum* subsp. *pertenue*	Guinea_Chimp_6117	Guinea	*Pan troglodytes*	Clinically acquired	Skin lesion		TGA 965-967 TGG	1	–	–	–
		1311	*T. pallidum* subsp. *pertenue*	SOL-3638288	Solomon Islands	*Homo sapiens*	Clinically acquired	Skin lesion		TGA 965-967 TGC	1	–	–	–
		1523	*T. pallidum* subsp. *pallidum*	ZIM015	Zimbabwe	*Homo sapiens*	Clinically acquired	Genital swab		TGA 965-967 AGC	1	–	–	–
RpsJ (30S ribosomal subunit protein S10)	GenBank	–	*Candidatus Borreliella tachyglossi*	–	–	–	–	–	1	V57A	1	–	–	–
		–	*Treponema porcinum*	–	–	–	–	–	2	V57G	2	–	–	–
		–	*Treponema* spp.	–	–	–	–	–	60	V57G	12	–	–	–
										V57I	1	–	–	–
										V57K	3	–	–	–
		–	*Borrelia persica*	–	–	–	–	–	1	V57I	1	–	–	–
		–	*Spirochaeta* spp.	–	–	–	–	–	1,006	V57G	1	–	–	–
										V57I	4	–	–	–
										V57K	17	–	–	–
		–	Other *Treponema* spp.	–	–	–	–	–	1,341	V57K	15	–	–	–
RpsC (30S ribosomal subunit protein S3)	GenBank	–	*Spirochaetales* bacterium	–	–	–	–	–	5	H178Q	4	–	–	–

–, Not available or not determined.

### Mutations in the *rpsJ* gene

Analysis of the *rpsJ* gene, which encodes the 30S ribosomal subunit protein S10, showed the presence of the tetracycline resistance-associated V57G substitution in both *Treponema porcinum* genomes and 20% of *Treponema* spp. genomes (Table 2 and Fig. 2). The presence of other variants, V57I and V57K, was observed at a lower prevalence (Table 2 and Fig. 2). The substitution V57K (169 GTG-AAG 171) frequently appeared in the *Spirochaeta* spp. (1.69% of genomes) and other *Treponema* spp. (1.12% of genomes; Table 2 and Fig. 2). The multiple sequence alignment of the *rpsJ* sequences is provided as File S2.

### Mutations in the *rpsC* gene

H178Q substitution in the *rpsC* gene that encodes the 30S ribosomal subunit protein S3 was found in 80% of the genomes of the *Spirochaetales* bacterium (Table 2 and Fig. 2). The multiple sequence alignment of the *rpsC* sequences is provided as File S3.

### MLST typing

Out of 4,355 genomes analysed, sequence types (STs) were identified for 178 genomes, with 145 classified as SS14-like and 33 as Nichols-like. No loci were found for 3,778 genomes. While 248 genomes contained allele information, they could not be assigned an ST type and hence were not typeable. MLST typing is provided in Table S1.

## Discussion

The resurgence of syphilis incidence globally necessitates innovative interventions to curb this trend [1]. As noted above, one promising intervention is doxy-PEP [5]. However, the extensive use of doxycycline for this purpose could select for tetracycline resistance in bacteria such as *T. pallidum* [10].

This study focused on the potential chromosomal mutations associated with tetracycline resistance within the *Spirochaetales* order. Our *in silico* analysis of 4,355 WGSs and an additional 613 16S rRNA sequences revealed the identification of a transition mutation from TGA to TGG/GGT/CGC/CGA at positions 965 to 967 (*E. coli* 16S rRNA numbering) in the 16S rRNA gene of *Treponema* and *Spirochaeta* spp. genomes.

Among the five *T. pallidum* sequences analysed from the PubMLST collection, the TGA to TGR (A/G) mutation was identified in two isolates of *T. pallidum* subsp. *pertenue* (IDs: 1584 and 1585). Additionally, TGA to TGG and TGC mutation in one isolate each of *T. pallidum* subsp. *pertenue* (IDs: 1582 and 1311, respectively) was identified. In *T. pallidum* subsp. *pallidum*, a TGA to AGC mutation was identified (Table 2). Further inspection of the full-length sequences of the five *T. pallidum* sequences showed the presence of degenerate nt bases. Notably, the TGG mutation present in isolate ID 1582 was identified in >1,000 isolates of other *Spirochetes* and Treponemes (Tables 2 and S2 and File S1a), suggesting that the mutation is unlikely to result from sequencing error and instead, the degenerate nt bases might arise from the sequencing of a heterogeneous population. The identification of this mutation across these species suggests a potential mechanism of resistance that might become more prevalent under selective pressure from increased doxycycline use [39]. Although less frequent, other mutations in this gene point to the potential for diverse resistance mechanisms within these genera.

In the *rpsJ* gene, the V57G aa substitution was present in both the genomes of *T. porcinum* and a significant subset of *Treponema* spp. This mutation is causally associated with reduced susceptibility to tetracyclines in a range of bacterial species [40]. While the V57G substitution in *rpsJ* has been functionally validated in other bacterial species, its role in *Treponema* spp. remains putative.

The V57I and V57K substitutions were also observed, but at lower frequencies. Notably, the GTG-AAG (V57K) mutation at nt positions 169–171 was relatively common in *Spirochaeta* spp. and other *Treponema* spp. Targeted mutagenesis studies have established that the V57K substitution results in hypersusceptibility to tetracycline in *E*. coli, whereas the V57I and V57L substitutions led to reduced tetracycline susceptibility [41]. Substitutions in V57 amino acid positions that confer resistance to tetracycline or tigecycline have been observed in both Gram-negative and Gram-positive bacteria, namely, *E. coli*, *Neisseria gonorrhoeae*, *Neisseria meningitidis*, *K. pneumoniae*, *Bacillus subtilis*, *Enterococcus faecium*, *Enterococcus faecalis* and *Staphylococcus aureus* [25,41,43].

The *rpsC* gene analysis uncovered an H178Q (corresponding to the H175 position in *S. pneumoniae*) substitution in a substantial majority of the *Spirochaetales* bacterium genomes. However, only five sequences were available for this analysis [24]. While similar mutations have been implicated in resistance in other bacterial species, there is no direct evidence linking these mutations to AMR in spirochetes. Additionally, the small sample size of available sequences, e.g. *rpsC* (*n*=5), and the lack of metadata for the genomes limit the generalizability of our findings. Therefore, *in vitro* studies, such as drug sensitivity testing to confirm the phenotype and clinical validation, are required to establish a causal relationship between these mutations and tetracycline resistance in *T. pallidum*.

The caveats of the study included the following: first, the 16S rRNA mutations observed in the *T. pallidum* (*n*=5) isolates were derived from the PubMLST isolate collection, and the WGSs of the isolates were unavailable, precluding a comprehensive analysis of the association between the presence of macrolide and tetracycline resistance genes. Second, our ability to evaluate the prevalence of resistance mutations by region and in relation to geographic doxycycline exposure was constrained by the limited metadata – particularly the absence of country-specific data on doxycycline consumption and implementation of doxy-PEP.

Of the 178 genomes for which MLST types were assigned, most were SS14-like (*n*=145) and a minority Nichols-like (*n*=33). Among these, the V57G *rpsJ* substitution did not show clear clustering by MLST lineage. However, as typeable genomes represented only ~4% of the total dataset, it constrains the analysis. Future studies with improved genomic coverage and lineage assignments could help clarify whether tetracycline-associated mutations are lineage-specific.

In addition, the use of metagenomic data may provide enhanced sensitivity for detecting resistance determinants. Nevertheless, in light of the widespread use of doxy-PEP, the putative mutations associated with tetracycline resistance reported in this study provide guidance for developing molecular methods for the rapid detection and surveillance of doxycycline-resistant *T. pallidum* in clinical specimens from doxy-PEP recipients [44]. We recommend cautious interpretation of these findings and emphasize the need for functional validation to confirm the role of these mutations in conferring tetracycline resistance in *T. pallidum*.

## Supplementary material

10.1099/acmi.0.000963.v4 Uncited Supplementary Material 1.

## Abbreviations

AMR: antimicrobial resistance
Barrnap: BAsic Rapid Ribosomal RNA Predictor
doxy-PEP: doxycycline post-exposure prophylaxis
HIV: Human Immunodeficiency Virus
MSM: men who have sex with men
WGS: whole-genome sequence
